# Supplementary material for: Exploration of Cytochrome P450-Related Interactions between Aflatoxin B1 and Tiamulin in Broiler Chickens
Source: Toxins (Basel). 2024 Mar 20;16(3):160. doi: 10.3390/toxins16030160 (PMC10974768; doi:10.3390/toxins16030160)
Supplement: Supplementary file 1 [file toxins-16-00160-s001.zip › toxins-2860076-supplementary.pdf]

Supplemental materials: Exploration of Cytochrome P450-Related Interactions between Aflatoxin B1 and Tiamulin in Broiler Chickens

Table S1. Aflatoxin B1 concentration (μg/kg) in mixed chicken feed

Sample name	Concentration (μg/kg)
Sample 1	9.30
Sample 2	15.70
Sample 3	15.10
Sample 4	15.60
Sample 5	10.90
Sample 6	13.70
Sample 7	10.90
Sample 8	13.30
Sample 9	16.50
Sample 10	13.50
Sample 11	15.70
Sample 12	17.90
Sample 13	13.10
Sample 14	21.70
Sample 15	12.70
Sample 16	11.90
Sample 17	13.20
Sample 18	25.40
Sample 19	13.40
Sample 20	15.50
Sample 21	23.20
Sample 22	16.00
Sample 23	13.10
Sample 24	13.70
Sample 25	12.00
Sample 26	11.90
Sample 27	13.10
Sample 28	12.90
Sample 29	17.30
Sample 30	1.20
Sample 31	14.00
Sample 32	12.80
Sample 33	12.90

Table S2. The final concentration of six probe substrates in the enzymes kinetic study

Analyte	Concentration (μM)
Phenacetin	4.5, 8.9, 22.3, 44.6, 89.3, 133.9, 178.6, 223.2
Bupropion	0.83, 1.7, 4.2, 8.3, 16.7, 25.02, 33.36
Chlorzoxazone	1.2, 2.4, 5.9, 23.6, 35.4, 47.2, 59.0
Tolbutamide	0.7, 1.5, 3.7, 7.3, 14.7, 58.4, 116.8
Coumarin	34.2, 68.4, 136.8, 205.2, 273.6, 342.0
Midazolam	0.6, 1.2, 2.5, 4.9, 12.3, 24.6, 49.1

Table S3. Mean (± SD) tiamulin (TIA) and aflatoxin B1 (AFB1) concentration (ng/mL) in chicken plasma after oral administration.

**Times (h)**	**TIA concentration (ng/mL)**		**AFB1 concentration (ng/mL)**	
	**Treatment group**	**Control group**	**Treatment group**	**Control group**
0.25	134 ±71.5	77.7±52.7	40.34±8.2	15.2±4.2
0.50	83.7±40.2	45.6±31.2	37.3±6.5	30.2±3.9
1	76.7±40.2	104±157.4	28.8±13.2	18.9±7.7
2	91.0±61.2	56.4±45.4	16.1±9.6	14.5±10.6
3	73.6±32.9	49.9±68.1	9.3±4.2	7.5±5.9
4	29.6±13.8	22.0±13.2	4.8±4.4	5.8±4.8
6	10.8±3.6	12.7±13.1	8.2±1.6	5.6±0.4
8	10.4±3.7	6.60±2.0	9.4±2	5.4±2
12	9.60±3.0	6.00±3.0	9.2±2.5	1.8±0.7
24	9.50±3.3	6.00±2.2	8.2±6.3	0.93±0.2

Instrument conditions for AFB1 and TIA analysis in plasma

AFB1 analysis: The plasma samples were examined by an UHPLC system (Thermo Fisher, USA) coupled to a TQS-MS (Thermo Fisher, USA). The separation of AFB1 was achieved through a HSS T3 column (100mm × 2.1mm, 1.7 μm) at the operating flow rate of 0.3 mL/min. Mobile phase A: aqueous solution containing 0.1% formic acid and mobile phase B: ACN. Gradient elution program was performed as follows: 0-0.5 min (5% B); 0.5-2.0 min (5% -95% B); 2.0-3.5 min (95% B); 3.5-4.0 min (95% -5% B); 4.0-5.0min (5% B). The injection volume was 5 μL. The system was operated in a positive electrospray ionization mode (ESI+) with the following typical parameters for mass spectrometry: spray voltage, 3.0 kV; Capillary temperature, 320 °C; Aux gas heater temperature, 350 °C; sheath gas flow rate, 40 arb; aux gas flow rate, 10 arb.

TIA analysis: Liquid chromatography was performed on an ACQUITY UHPLC system (Waters, Milford, USA) coupled with a XEVO TQS triple quadrupole tandem mass spectrometer (Waters, Milford, USA). The mobile phase comprised solution A (0.1% formic acid in water) and solution B (0.1% formic acid in methanol). The gradient elution program was performed as follows: 0-1.0 min (10% B); 1.0-2.5 min (10% -90% B); 2.5-3.0 min (90% B); 3.0-4.0 min (90%-10% B); 4.0-5.0 min (10% B). The injection volume was 5 μL and the flow rate was 0.3 mL/min. An Acquity HSS T3 C18 column (100 mm × 2.1 mm, 1.7 μm) was used to perform the analysis at a temperature of 40°C.

The mass spectrometer contained an electrospray ionization (ESI) source. The ESI source was operated in positive ionization mode with capillary voltage, 3.0 kV; source temperature, 120 °C;

desolvation temperature, 500 °C; desolvation gas flow rate, 100 L/h; and cone gas flow rate, 150 L/h.

Example chromatograms of TIA and AFB1 and of CYP450 probes

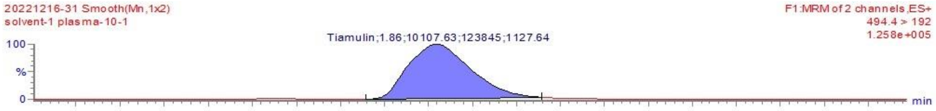

Figure S1. Example chromatogram of tiamulin in broiler chicken plasma

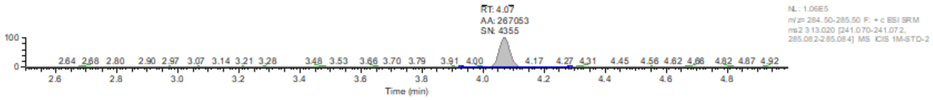

Figure S2. Example chromatogram of aflatoxin B1 in broiler chicken plasma

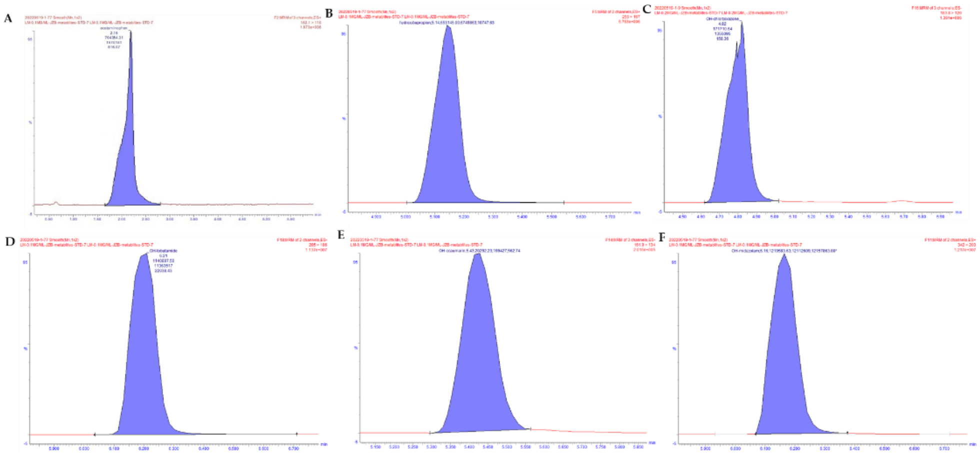

Figure S3. Example chromatograms of the different probes of CYP450 enzymes: (A) ccetaminophen; (B) OH-bupropion; (C) OH-chlorzoxazone; (D) OH-tolbutamide; (E) OH-coumarin; and (F) OH-midazolam.
